# The Effect of Nutritional Education on Nutritional Status and Quality of Life in Patients with Liver Cirrhosis

**DOI:** 10.3390/healthcare13151905

**Published:** 2025-08-05

**Authors:** Seymanur Tinkilic, Perim Fatma Turker, Can Selim Yilmaz, Meral Akdogan Kayhan, Derya Ari, Dilara Turan Gökce

**Affiliations:** 1 Department of Nutrition and Dietetic, Baskent University, Ankara 06790, Türkiye; seymanur.tinkilic@gmail.com (S.T.); csyilmaz@baskent.edu.tr (C.S.Y.); 2 Department of Nutrition and Dietetics, Acibadem Mehmet Ali Aydinlar University, Istanbul 34752, Türkiye; 3 Department of Gastroenterology, Bilkent City Hospital, Ankara 06700, Türkiye; akdmeral@yahoo.com (M.A.K.); deryaari81@hotmail.com (D.A.); dilaraturan89@yahoo.com (D.T.G.)

**Keywords:** liver cirrhosis, nutritional education, nutritional status, quality of life, dietary habits

## Abstract

*Objectives:* This study aimed to evaluate the effect of nutritional education on nutritional knowledge, nutritional status, and quality of life in patients with liver cirrhosis. *Methods:* Thirty patients participated. At baseline, assessments were conducted to collect data on demographics, physical activity, anthropometric and biochemical measures, dietary habits, 24 h food intake, nutritional status, quality of life, and nutritional knowledge. Participants received a 30 min face-to-face nutritional education session by a registered dietitian, repeated after one month. A follow-up phone call was conducted one month later to reinforce the education. Final evaluations were completed one month after the call. *Results:* A significant upward trend was detected in nutritional knowledge scores after the intervention period (from 7.4 ± 2.76 to 9.2 ± 3.45). The physical component of quality of life improved, while the mental component showed a slight decline. Dietary changes included reduced energy and protein intake among females and increased protein intake in males. In both genders, fat intake increased and carbohydrate intake decreased. Biochemical improvements were observed, including significant reductions in gamma-glutamyl transferase, aspartate aminotransferase, alanine aminotransferase, and triglycerides in females and alanine aminotransferase and gamma-glutamyl transferase in males. *Conclusions:* Structured nutritional education may improve nutritional knowledge, dietary behavior, and biochemical markers in cirrhosis patients. Longer follow-up durations may further enhance these improvements.

## 1. Introduction

Liver cirrhosis, defined by hepatocellular degeneration, fibrosis, and regenerative nodules leading to hepatic dysfunction, is a major global health concern [1,2,3]. It significantly contributes to morbidity and mortality associated with chronic liver disease. In 2019, cirrhosis accounted for 2.4% of global deaths, and by 2023, liver-related conditions (primarily cirrhosis and hepatocellular carcinoma) were responsible for approximately two million deaths annually, representing 4% of all global mortality. The majority of these deaths were observed among males [4,5].

Despite the complexity of managing cirrhosis, an organized, multidisciplinary, and systematic approach is essential. Malnutrition is a common and serious complication in cirrhotic patients, adversely impacting disease prognosis by exacerbating conditions such as sarcopenia, hepatic encephalopathy, and susceptibility to infections [6]. It is associated with higher rates of complications, including sepsis, ascites, spontaneous bacterial peritonitis, and hepatorenal syndrome, affecting up to 65% of malnourished patients compared to 12% of those well-nourished [7]. Malnutrition and sarcopenia in cirrhosis are associated with reduced survival rates, decreased quality of life (QoL), increased risk of complications, and worse postoperative and post-transplant outcomes. However, the diagnosis and management of malnutrition in this population remain suboptimal. Reported prevalence rates of malnutrition in cirrhosis range widely from 5% to 92%, likely due to limited awareness, lack of medical and nutritional education, and challenges in accurate assessment [6,7,8].

Nutritional education is a comprehensive process that fosters practical, cognitive, and emotional awareness. In the context of chronic disease management, effective nutritional education interventions have been shown to enhance patients’ knowledge, which serves as a foundational step toward adopting healthier, disease-appropriate lifestyle behaviors. Improved disease-specific knowledge can contribute to better symptom management, reduced hospitalization rates, and enhanced self-regulation in individuals living with chronic conditions [9]. The complexity of liver cirrhosis and its complications often impairs patients’ ability to access and comprehend disease-related information. Additionally, limited consultation time in routine clinical settings restricts physicians’ capacity to provide comprehensive nutritional education [10]. Clinical practice guidelines highlight the necessity of individualized nutritional education and dietary counseling by qualified nutrition professionals, such as dietitians and nutritionists, for patients with cirrhosis [11,12]. Evidence suggests that these patients frequently experience inadequate energy and protein intake and poor overall diet quality, falling short of nutritional recommendations [13,14,15,16]. Furthermore, patients with liver cirrhosis commonly encounter challenges in nutritional management, including limited access to a dietitian, frequent misconceptions regarding dietary recommendations, and poor adherence to recommendations [17,18]. Previous research in patients with cirrhosis has highlighted that nutritional education interventions are associated with significant improvements in clinical outcomes (i.e., reductions in ascites and edema, enhancement in QoL). These approaches have demonstrated promising results and underscore the need for further investigation to validate their efficacy and long-term impact [10,19].

Multiple studies have highlighted the positive impact of nutritional education interventions and continuous follow-up on clinical outcomes and QoL in patients with cirrhosis. One such study demonstrated that face-to-face nutritional education followed by a six-week monitoring period resulted in significant improvements in ascites, edema, patient knowledge, and QoL, alongside reductions in hepatic encephalopathy (HE), variceal bleeding, and hospitalization rates [10]. Similarly, in a randomized controlled trial, a six-month nutrition education program significantly improved health-related QoL and decreased HE scores compared to controls [20]. Additionally, the implementation of a nutritional guideline over six months was found to significantly enhance patients’ nutritional knowledge, although no substantial changes were observed in QoL or malnutrition risk [19].

Studies involving diverse patient populations, including those with obesity, diabetes, multiple sclerosis, psychiatric disorders, and kidney disease, have demonstrated that nutrition educational interventions can lead to improvements in nutritional knowledge, QoL, and nutritional status [21,22,23]. Although patient education is considered a key component of supportive care in liver cirrhosis, evidence is lacking on the baseline knowledge of the patients regarding the disease and the effectiveness of structured nutritional education programs.

This study aimed to evaluate the effect of nutrition education on QoL, nutritional knowledge, and nutritional status in individuals with liver cirrhosis. Accordingly, the present study hypothesized that a structured nutrition education intervention would lead to improvements in patients’ nutritional knowledge, facilitate positive changes in dietary intake patterns and select biochemical parameters, and contribute to an overall enhancement in QoL.

## 2. Materials and Methods

### 2.1. Study Population

This study employed a longitudinal observational design to evaluate the effects of nutritional education on patients with liver cirrhosis across several parameters, including nutritional status, quality of life, and biochemical markers.

This observational follow-up study was conducted on 30 adults who presented to the gastroenterology polyclinic of a local hospital in Ankara, Türkiye, between December 2021 and June 2024. Inclusion criteria comprised individuals aged 18 to 59 years, diagnosed with liver cirrhosis by a gastroenterologist within the past five years, without sensory impairments (e.g., hearing or vision loss) that could hinder participation in nutritional education sessions, and who consented to attend clinic-based nutrition education sessions. Patients in the intensive decompensated phase of cirrhosis (Child–Pugh class C), those who were pregnant or lactating, individuals with prior structured nutrition education, and those receiving oral nutritional supplements or any form of nutritional support were excluded. Additional exclusions comprised individuals with a history of alcohol consumption, infectious diseases, malignancies, hepatic encephalopathy, prior liver transplantation, hepatocellular carcinoma, hepatorenal syndrome, or conditions affecting nutrient absorption (e.g., celiac disease). Patients with chronic comorbidities such as diabetes, renal disorders, and Wilson’s disease were also excluded.

Ethical approval for this study was obtained from the Ethics Committee of Baskent University (Approval number: KA21/216). Written informed consent was obtained from all participants prior to enrolment. All procedures involving human participants were performed in accordance with the ethical standards of the institutional and/or national research committees and the tenets of the 1964 Declaration of Helsinki and its subsequent revisions or equivalent ethical guidelines.

The sample size calculation for this study was performed using power analysis with G*Power (version 3.1.9.2) with an effect size of 0.5, 80% power, and 0.05 error margin. The results indicated that a minimum sample size of 30 participants would be required. During the data collection phase, a total of 45 individuals (17 females and 28 males) were initially screened in the study. However, the final analysis included 30 participants (9 females and 21 males). Fifteen individuals were excluded for the following reasons: non-attendance at the nutritional education sessions (*n* = 9), subsequent diagnosis of cancer (n = 2), diagnosis of a condition necessitating a specialized diet (*n* = 1), and undergoing liver transplantation during the study period (*n* = 3).

### 2.2. Nutrition Education

Previous studies on nutritional education interventions in cirrhotic patients [10,19] often employed a combination of counseling and guidebooks, resulting in improvements in clinical symptoms despite limited changes in biochemical parameters. Building upon this methodology, the present study implemented a nutrition education program comprising face-to-face sessions with a dietitian and a validated counseling booklet. Among existing tools, the Thai counseling booklet [17] is recognized as the most rigorously validated nutritional education resource for cirrhosis. Although other programs generally adhere to guideline-based content—including disease education, nutritional goals, follow-up protocols, and meal planning strategies—they frequently lack formal validation. Therefore, the Turkish version of the Thai booklet was used for this study. The dietitian-led sessions incorporated this booklet along with a PowerPoint presentation developed in accordance with evidence-based national and international nutritional guidelines such as the Turkey Dietary Guidelines (TUBER) [24] and those of the European Society for Clinical Nutrition and Metabolism (ESPEN) [18] and the European Association for the Study of the Liver (EASL) [25].

The content presented through the PowerPoint slides and accompanying booklet during face-to-face dietitian consultations provides a comprehensive overview of the pathophysiology of liver cirrhosis, with a particular focus on its impact on digestion, metabolism, and nutritional requirements. The primary objective is to enhance patients’ and caregivers’ understanding of the disease process and its nutritional implications. The nutritional education materials address common complications associated with cirrhosis, including sarcopenia, impaired wound healing, increased susceptibility to infections, ascites, and peripheral edema. Emphasis is placed on the importance of early and proactive dietary intervention, highlighting the established association between malnutrition and adverse clinical outcomes. Furthermore, the materials offer evidence-based recommendations on sodium restriction (particularly in the context of ascites), the maintenance of adequate hydration, and the selection of high-quality protein sources to support overall nutritional status and clinical stability. The program also included guidance on food safety practices to prevent infections, nutritional education on the prevention of nutrition-related complications, and information about food-drug interactions that could impact treatment outcomes [8,17,19].

**Figure 1:**
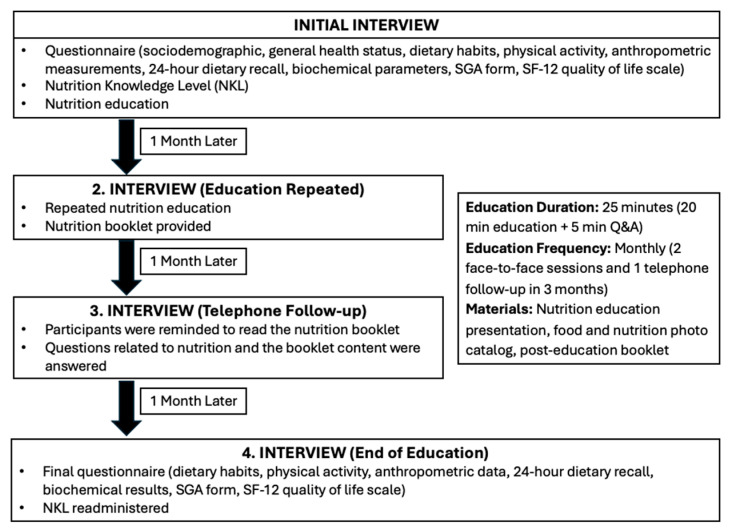
Study process flowchart.

The nutrition education was delivered at the initial interview and repeated one month later in a second face-to-face session. Additionally, a telephone follow-up interview was conducted one month after the second session, in which participants were reminded to read the nutrition booklet and were asked questions about its content. The flowchart of the study is detailed in Figure 1.

The nutritional education intervention implemented in this study was not integrated into the standard clinical care of the participating patients but was carried out solely for research purposes. Accordingly, the study was designed as a nutritional education intervention specifically intended to investigate its effects within a research framework.

Patient cooperation throughout the nutritional education intervention involved several key responsibilities: consistent attendance at nutritional education sessions, thorough review of provided materials, completion of assigned tasks (e.g., food intake records), practical application of recommended nutritional behaviors, and participation in follow-up evaluations, including telephone check-ins. Compliance with the intervention was evaluated using multiple measures, including biochemical parameters (blood results), maintenance of food and medication intake diaries, responsiveness to follow-up calls or messages, and self-reported adherence. Participants who consistently met the study objectives and predefined criteria across these measures were evaluated as compliant.

### 2.3. Participant Demographics and Anthropometric Measurements

The socio-demographic data were collected at the initial face-to-face interviews through a structured questionnaire. Anthropometric measurements, including height, body weight, mid-upper-arm circumference, and triceps skinfold thickness, were obtained by the researchers at both baseline and post-intervention using standardized procedures. Height was measured in centimeters with a calibrated Seca stadiometer and body weight in kilograms using a digital scale, with Body Mass Index (BMI) subsequently calculated (kg/m^2^). Mid-upper-arm circumference was measured at the midpoint between the acromion and olecranon processes with the arm relaxed after marking the location with the arm flexed at 90 degrees. Triceps skinfold thickness was assessed using a caliper at the same midpoint on the posterior right arm, also with the arm flexed at 90 degrees.

### 2.4. Assessment of Food Consumption Status

Data on food consumption of patients were gathered through a 24 h dietary recall method (conducted on three consecutive days of the week following the end of treatment and done on the basis of three separate conversations). Daily food consumption and energy intake were evaluated using the Nutrition Information System (BeBis 8.1 version) by the researchers. Average dietary intake was calculated by the researchers using three-day food records. Micronutrient intakes were analyzed and evaluated in accordance with age- and gender-specific Dietary Reference Intake (DRI) guidelines [26].

### 2.5. Evaluation of Biochemical Findings

The biochemical parameters evaluated in this study were those routinely ordered by the attending gastroenterologist during clinical examinations and follow-up visits. Blood samples were analyzed at the biochemistry laboratory of the hospital, and reference values were obtained from the same institution. Participants were instructed to fast for a minimum of eight hours prior to morning blood collection. Biochemical assessments were conducted at two time points: one week prior to the initial nutrition education session and within one week after the end of the three-month nutritional education.

### 2.6. Assessment of Nutritional Status

The Subjective Global Assessment (SGA) is a practical, time-efficient, and cost-effective tool commonly employed to evaluate nutritional status and determine the severity of malnutrition. Given the high prevalence of malnutrition among patients with liver cirrhosis—and its recognized role as a predictor of disease progression—clinical guidelines, including ESPEN and EASL, recommend the use of SGA in this patient population [27]. The nutritional status was assessed using the SGA tool, which categorizes individuals into three groups: A (well-nourished), B (moderate or suspected malnutrition), and C (severe malnutrition) [28]. In this study, the SGA was administered at two time points: prior to the initial nutritional education session and during the final evaluation conducted three months later.

### 2.7. Evaluation of Quality of Life (QoL)

The 12-item short-form health survey (SF-12), developed by Ware and Sherbourne [29], is a self-report instrument designed to assess the QoL. In the present study, the validated Turkish version of the SF-12 was used [30]. The SF-12 QoL questionnaire comprises eight subgroups. The Physical Component Summary (PCS-12) score is derived from the general health, physical functioning, role-physical, and bodily pain subgroups, whereas the Mental Component Summary (MCS-12) score is calculated based on social functioning, role-emotional, mental health, and vitality subgroups. Both the PCS-12 and MCS-12 scores range from 0 to 100, with higher scores indicating a better QoL [31]. In this study, the SF-12 scale was administered at two time points: prior to the initial nutritional education session and at the final assessment conducted three months later.

### 2.8. Determination of Nutritional Knowledge Level (NKL)

A Nutritional Knowledge Level (NKL) assessment form was developed by the researchers using data from previous studies [24,32,33,34,35], incorporating general nutrition concepts, liver cirrhosis-specific content, and dietary recommendations. The form comprised 20 multiple-choice questions, with each correct answer scored as 1 point and incorrect answers as 0. Total scores were used to classify knowledge levels as low (≤5), moderate [6,7,8,9,10,11,12,13,14,15], or high (≥15) based on percentile cutoffs. The NKL form was administered twice: prior to the initial nutrition education session and at the final assessment three months later.

### 2.9. Statistical Analysis

The data obtained in the study were analyzed using IBM SPSS Statistics version 25.0. Descriptive statistics such as mean ($\bar{X}$), standard deviation (±SD), number (n), and percentage (%) were calculated. To determine the appropriate statistical analyses, the Shapiro–Wilk test (applicable for sample sizes ≤ 30) was initially conducted to assess the normality of all measurement distributions. The results indicated that the data conformed to the assumption of normal distribution; consequently, parametric tests were employed for subsequent comparisons. Parametric tests were used for normally distributed variables, while the Mann–Whitney U test was applied for non-normally distributed data. The paired samples *t*-test assessed pre- and post-intervention differences in continuous variables. McNemar’s test evaluated changes in categorical variables over time. Associations between categorical variables were examined using the chi-square test; where its assumptions were not met, Fisher’s exact test (for 2 × 2 tables) and the Freeman–Halton test (for larger tables) were employed. Pearson’s correlation coefficient was used to assess relationships between continuous variables. All statistical analyses were two-tailed, and a *p*-value of <0.05 was considered statistically significant.

## 3. Results

The socio-demographic characteristics of the participants are shown in Table 1. The study sample consisted of 30 patients (mean age 45.1 ± 10.88 years) undergoing SG, of whom 70.0% (n = 21) were female and 30.0% (n = 9) were male. The mean BMI of the patients at baseline and after intervention was 26.6 ± 5.03 and 27.1 ± 5.22, respectively (not statistically significant different between genders). No significant difference was observed in mid-upper-arm circumference and triceps skinfold thickness between baseline and following the intervention, nor between genders. No statistically significant differences were identified between genders in terms of nutritional education attainment, etiology of liver cirrhosis, presence of comorbidities, or engagement in regular physical activity. The further socio-demographic characteristics of patients are shown in Table 1.

The biochemical findings of the participants before and following the nutritional education are presented in Table 2. Females exhibited significant reductions in gamma-glutamyl transferase (GGT), aspartate aminotransferase (AST), alanine aminotransferase (ALT), and triglyceride levels (*p* < 0.05), whereas significant reductions were observed in ALT and GGT levels in males (*p* < 0.05). Only triglyceride levels showed a significant difference between genders. No statistically significant differences were observed in other biochemical parameters before and after the intervention in either gender (*p* > 0.05).

The total energy and macronutrient intakes of participants before and following the nutritional education are represented in Table 3. Following the nutritional education, a reduction in energy (from 20.9 ± 11.19 kcal/kg to 16.9 ± 11.40 kcal/kg) and protein intake (0.6 ± 0.38 g/kg to 0.5 ± 0.31 g/kg) were observed in females; however, in male participants, an increase in protein intake (0.7 ± 0.56 g/kg to 0.8 ± 0.40 g/kg) was detected. Both genders showed an increase in the proportion of energy derived from fat and a reduction in dietary carbohydrate intake (both are significant in females).

Micronutrient intakes of participants before and following the nutritional education intervention are presented in Table 4. Regarding micronutrient intake, no statistically significant differences were found between before and after nutritional education measurements in either gender (*p* > 0.05). Among the assessed micronutrients, the proportion of RDI met varied between 40.1% and 180.4%. Notably, niacin, riboflavin, folic acid, potassium, calcium, and magnesium demonstrated lower RDI fulfillment rates compared to other micronutrients.

Classification using the Subjective Global Assessment of individuals is shown in Table 5. The number of patients scored as B or C according to SGA decreased after the nutritional education in both genders (B: from 53.3% to 36.7%; C: from 10% to 3.3%), but no statistical significance was observed among the results.

The QoL and nutritional knowledge level scale scores of individuals before and following the nutrition education are represented in Table 6. Following the three-month intervention, a non-significant improvement was observed in the physical component summary (PCS-12) score of QoL in female participants. In contrast, the mental component summary (MCS-12) score demonstrated a slight decline (from 50.9 ± 11.64 to 46.3 ± 9.49), which reached statistical significance among males. Nutritional knowledge scores significantly increased overall (from 7.4 ± 2.76 to 9.2 ± 3.45), with a statistically significant improvement observed in females and a positive trend noted in males. Prior to the nutritional education intervention, males demonstrated higher levels of nutritional knowledge; however, following the intervention, females exhibited greater improvement, resulting in higher post-intervention knowledge scores.

## 4. Discussion

Dietary management constitutes a fundamental component in the clinical management of patients with cirrhosis, playing a pivotal role in optimizing health outcomes and mitigating disease-related complications. Early nutritional interventions may positively influence the progression of the disease. Moreover, the early presence of a dietitian in the healthcare group, to assess and manage nutritional risks in these patients, was shown to provide better survival outcomes [36]. This study aimed to evaluate the impact of nutrition education on QoL, nutritional knowledge, and nutritional status in individuals with liver cirrhosis. In this study, following the intervention period, a significant increase was observed in nutritional knowledge scores. While the physical dimension of health-related QoL showed improvement, a slight decline was noted in the mental dimension. In terms of dietary intake, females demonstrated reductions in total energy and protein consumption, whereas protein intake increased among males. Additionally, both genders exhibited an increase in fat intake and a decrease in carbohydrate consumption. Biochemical analyses revealed favorable changes, including significant reductions in gamma-glutamyl transferase, aspartate aminotransferase, alanine aminotransferase, and triglyceride levels in females, and alanine aminotransferase and gamma-glutamyl transferase levels in males.

This study demonstrated a statistically significant enhancement in participants’ nutritional knowledge scores following the nutrition education intervention when compared to before. In the context of chronic disease management, evidence from the literature, similar to our results, indicates that well-structured educational interventions significantly enhance individuals’ knowledge and function as a critical catalyst for promoting behavioral change [9,10,19,21,22,37,38]. Increased knowledge is anticipated to facilitate the adoption of healthier lifestyle practices aligned with the management of the disease. Educating patients about their condition has been associated with reduced hospitalization rates, improved disease-specific symptoms, and enhanced self-management capabilities. A narrative review covering the period from 2000 to 2023 outlined various educational strategies employed to support patients with cirrhosis, including printed materials and technology-assisted tools integrated with routine dietary counseling. While the available evidence remains limited, these methods have demonstrated promising outcomes and underscore the need for further research [19]. A quasi-experimental pilot study (n = 72) assessed the impact of a six-month intervention comprising nutritional counseling, nutritional education booklets, and regular monitoring. Although no significant changes were observed in biochemical parameters, the intervention led to notable improvements in clinical outcomes, including reductions in ascites and edema, as well as a significant enhancement in QoL [10]. Nutritional education has shown positive effects across various clinical populations. In diabetic patients, while nutrient intake remained unchanged, improvements were noted in nutrition knowledge and dietary behavior [21]. Hemodialysis patients experienced enhanced QoL and increased nutrition knowledge following nutritional education [22]. Psychiatric patients showed reduced energy and carbohydrate intake, greater intake of vitamin C and iron, lower depression and anxiety levels, and improved nutritional awareness [37]. In multiple sclerosis patients, although not statistically significant, positive trends were observed in sleep quality, physical activity, diet quality, mood, and nutritional knowledge after the intervention [38].

The literature includes studies indicating that liver cirrhosis negatively impacts QoL, while nutrition education interventions are associated with improvements in QoL among affected patients [10,22,39,40]. In the present study, although an improvement was observed in the physical component of QoL, a slight decline was noted in the mental component. Overall, these changes did not result in a statistically significant difference in total QoL scores, in contrast to findings reported in the literature. These results may be attributed to the relatively short duration of the intervention and the limited sample size employed in the study.

In this study, post-nutritional education meal frequencies increased slightly to an average of 2.75 main meals and 1.63 snack meals per day, but the changes were not statistically significant. The number of participants not consuming snacks decreased slightly. Nutrition guidelines for individuals with cirrhosis emphasize minimizing fasting periods and encouraging frequent meals. ESPEN recommends 3–5 meals per day (Level B evidence) [18], while EASL advises three main meals and three snacks daily [25]. Previous studies have shown that individuals with compensated cirrhosis consume an average of 2–3 meals per day, which is in line with our findings [13,14].

Sodium restriction is essential for managing fluid retention in cirrhosis, with a recommended limit of 5 g/day of salt [25,41,42,43]. Excessive sodium intake (>10 g/day of salt) is linked to multiple adverse health effects, while overly restrictive intake (<3 g/day of salt) may impair nutrient absorption and renal function [44,45]. In this study, participants were educated on limiting salt intake, and a non-significant reduction was observed post-intervention, considered potentially clinically meaningful. Following the training, a non-significant decrease was noted in individuals with normal and high salt intake, while a non-significant increase was observed in those reporting low salt intake.

Standard fluid requirements of 30–40 mL/kg/day apply unless serum sodium falls below 130 mEq/L, in which case fluid restriction is warranted [34,46]. Since participants with severe ascites were excluded from this study, no fluid restriction was applied. Mean daily water intake increased from 1736.7 ± 1038.06 mL before the nutritional education to 1818.3 ± 921.71 mL after the education; however, this change was not statistically significant.

In this study, the proportion of individuals who reported using frying as a cooking method decreased from 9.5% prior to the intervention to 4.2% following nutrition education. The nutritional education sessions promoted healthier cooking techniques, resulting in an increased preference for methods such as boiling and baking, while the use of less healthy methods like roasting (significant change, *p* < 0.05) and frying declined. Given the impact of cooking methods on lipid oxidation and the degradation of various micronutrients [47,48,49], the observed shift away from frying and roasting (methods associated with higher formation of oxidation products) represents a meaningful improvement in dietary practices.

Individuals with chronic liver disease frequently experience symptoms that impair eating ability, contributing to weight loss, malnutrition, and reduced QoL. Studies have shown that a significant proportion of patients report multiple nutrition-related symptoms, with common complaints including dry mouth, abdominal pain, diarrhea, and nausea. Malnourished individuals tend to report more symptoms and lower QoL. Gastrointestinal issues such as indigestion and constipation are prevalent in patients with chronic liver disease. Additionally, symptom severity may influence readiness to adopt healthier eating habits [39,40,50,51,52]. In the present study, 60% of participants reported at least one nutrition-related symptom before the nutritional education intervention, decreasing to 50% afterward. Although appetite scores slightly declined post-intervention, the change was not significant. In this study, similar to previous studies in the literature [51,53,54], abdominal bloating and gas were the most common symptoms both before and after the nutritional education. These symptoms may contribute to early satiety and pose challenges to achieving adequate nutritional intake.

Muscle wasting is a prevalent manifestation of malnutrition in individuals with cirrhosis and is associated with adverse clinical outcomes [55]. Anthropometric assessments of the upper extremities, such as triceps skinfold thickness and upper-arm circumference, serve as reliable indicators of nutritional status and sarcopenia, particularly due to their minimal susceptibility to fluid retention [56]. In a study conducted by Santos et al. [57], upper-arm muscle circumference was identified as a significant predictor of sarcopenia, whereas BMI was not. Furthermore, elevated upper-arm height ratio values have been linked to decreased mortality risk in patients with cirrhosis [55]. In the current study, a substantial proportion of participants (particularly males) had upper-arm circumferences below the age-specific 50th percentile both before and after the intervention, suggesting a heightened risk of future malnutrition. Concurrently, 30.1% of participants pre-intervention and 36.6% post-intervention were classified as obese, raising the possibility of sarcopenic obesity. This observation is supported by the absence of significant ascites or edema and suboptimal energy and protein intake. These findings align with similar trends reported in earlier studies [58,59,60] involving individuals with cirrhosis.

Biochemical assessments are commonly employed to evaluate the nutritional status of individuals. In a prior educational intervention targeting individuals with cirrhosis, significant improvements were reported in QoL, knowledge levels, duration of hospitalization, and clinical symptoms such as ascites and edema; however, these improvements were not reflected in biochemical parameters [10]. In contrast, nutrition education programs implemented among hemodialysis patients have demonstrated positive effects on both biochemical markers and certain complications [9,61,62]. Previous research has indicated that serum albumin levels are typically lower in cirrhotic patients with malnutrition [63,64,65]. Although changes in biochemical parameters were not statistically significant in the present study, the slight increases observed in both albumin and total protein levels may nonetheless suggest a trend toward improved nutritional status. In this study, a significant reduction in serum AST, ALTm, and GGT levels was observed among female participants following the nutritional education intervention, while in male participants, significant decreases were noted in ALT and GGT levels. GGT levels may be elevated in various liver diseases, regardless of their underlying etiology. Elevated GGT is observed in over 50% of individuals with non-alcoholic fatty liver disease (NAFLD) and approximately 30% of those with hepatitis C virus (HCV) infection, often reaching levels two to four times above the upper reference limit [66]. In a study by Nanri et al. [67], GGT levels were inversely associated with a dietary pattern characterized by high intake of fruits, vegetables, and fish, and positively associated with a pattern marked by increased consumption of bread and sweets. Similarly, Lorzadeh et al. [68] reported that GGT levels were adversely affected in healthy individuals adhering to a western dietary pattern, which is typically high in processed foods and low in fruits and vegetables. These reductions represent noteworthy outcomes of the intervention. It is hypothesized that these improvements may be attributable to dietary modifications, including reduced use of cooking methods associated with high levels of lipid oxidation products (e.g., frying and roasting), changes in the types of oils used, and/or increased intake of fruits and vegetables. In this study, the observed increases in serum total cholesterol and LDL-C (not significant), alongside a significant reduction in triglyceride levels among females, are likely attributable to increased dietary fat intake and reduced carbohydrate consumption. The observed reduction in total energy intake among females, in contrast to the stable total intake among males, may have contributed to a more rapid depletion of glycogen stores and a subsequent increase in lipolysis.

Oral intake in individuals with liver cirrhosis may be compromised due to factors such as early satiety, anorexia, dysgeusia, inadequate dietary intake and absorption, prescribed dietary restrictions, and certain medications [69]. Studies have shown that approximately 85–95% of individuals with cirrhosis have insufficient daily intake of total energy, protein, and carbohydrates, while nearly 50% demonstrate inadequate consumption of lipids and carbohydrates. These findings underscore the importance of implementing effective nutritional education programs to address these deficiencies and support dietary management in this population [70]. In this study, energy intake among males remained stable following the nutritional education intervention, whereas females exhibited a significant reduction in energy intake per kilogram of body weight. Protein intake per kilogram decreased significantly in females but increased significantly in males. Although cholesterol intake rose in females and declined in males, these changes were not statistically significant. Both genders demonstrated increases in total dietary fat and the proportion of energy derived from total fat, saturated fatty acids (SFA), monounsaturated fatty acids (MUFA), and polyunsaturated fatty acids (PUFA); however, only the increase in total fat percentage among women reached statistical significance. Carbohydrate intake declined in both groups, with a significant decrease observed in females. Fiber intake decreased in females and increased in males, though these changes were not statistically significant. Following the nutritional education intervention, analysis of micronutrient intake revealed an overall increase in the daily consumption of vitamin A, vitamin E, riboflavin, folate, vitamin B12, vitamin C, potassium, and calcium, alongside a decrease in the intake of niacin, phosphorus, iron, magnesium, and copper. Intake levels of thiamine, vitamin B6, and zinc remained unchanged. When micronutrient adequacy was assessed according to DRI standards, a reduction in the prevalence of inadequate intake was noted for vitamin A, riboflavin, folate, vitamin B12, vitamin C, calcium, zinc, and magnesium. Conversely, inadequacy increased for vitamin E, thiamine, niacin, vitamin B6, and potassium, while phosphorus and iron inadequacies remained unchanged. These differences were not statistically significant.

SGA is a widely utilized method for evaluating nutritional status in individuals with liver cirrhosis, despite its limited sensitivity. It is endorsed by both ESPEN and EASL for the nutritional assessment of patients with liver disease [18,25]. In the present study, the number of individuals classified as SGA A (well-nourished) increased following the nutritional education intervention, while the number of individuals classified as SGA B (moderately malnourished) and SGA C (severely malnourished) decreased. However, these changes were not statistically significant. The observed improvements may be attributed to a reduction in nutrition-related symptoms and a decrease in edema, possibly due to reduced salt intake. Additionally, as the study population consisted primarily of individuals in the early stages of cirrhosis, the relatively low prevalence of malnutrition aligns with findings reported in the literature [56]. Nevertheless, given the progressive nature of liver disease, early screening for anthropometric indicators and nutritional risk is essential for the timely identification and management of malnutrition.

### Strengths and Limitations

Although patient education is widely recognized as an essential part of supportive care for liver cirrhosis, there are still limited data on patients’ baseline knowledge of the disease and the effectiveness of structured educational programs. Therefore, focusing on the nutrition education in patients with liver cirrhosis may provide a valuable perspective and help to address the existing gap in the literature.

This study has a number of limitations that may guide future research designs. The literature lacks clearly defined indicators that directly reflect the response of patients with cirrhosis to nutrition education interventions. A review of comparable studies on nutrition education interventions demonstrates that, in alignment with the present study, evaluations typically focus on changes in biochemical markers, nutritional knowledge and health literacy, QoL, and dietary intake and eating behaviors, as well as anthropometric outcomes. Therefore, an important limitation is that changes in the indicators monitored in our study may be influenced by other factors. The absence of a control group represents a significant limitation, as it restricts the ability to draw definitive conclusions from the observed outcomes. Furthermore, implementing longer-term educational interventions may offer greater insight into the extent of change in monitored parameters and their potential divergence from established long-term trends. Longer-duration prospective studies with larger sample sizes in individuals with liver cirrhosis would provide evidence to assess the long-term effects of nutritional education. Increasing the frequency of educational sessions could have enhanced the overall effectiveness of the nutritional education intervention. Although the three-day dietary recall assessment is suitable for determining nutrient intake, its inherent susceptibility to recall and reporting bias is a potential limitation. The study was single-center, and some variables that may affect the effectiveness of the education, such as daily eating habits, physical activity levels, and nutrient–drug interactions during nutritional education interventions, were not considered.

## 5. Conclusions

The findings of this study suggest that a three-month nutrition education intervention in patients with liver cirrhosis may be associated with beneficial changes across multiple domains. The nutrition education intervention appears to be associated with notable improvements in several domains, including the frequency of daily snack consumption (non-significant increase), salt (non-significant reduction among participants reporting normal and high amounts of salt consumption), and water intake (non-significant increase). Additionally, nutritional education intervention may be associated with positive changes in preferred cooking methods (e.g., significant decrease in roasting method), nutrition-related symptoms (non-significant decrease), indicators of malnutrition (non-significant increase in the number of individuals classified as SGA A), and levels of nutrition knowledge (significant increase). Furthermore, while the intervention may contribute to improvements in dietary intake, it is associated with significant reductions in several biochemical parameters—such as GGT, AST, ALT, and triglycerides in females, and ALT and GGT in males—as well as improvements in QoL measures. In particular, the increase in PCS is higher in females, while the decrease in MCS is significantly greater in males.

These findings underscore the importance of systematically monitoring nutritional knowledge in this patient population, with particular attention to gender-specific differences. Supportive strategies, including individualized dietary counseling and educational interventions aimed at regularly assessing and enhancing nutritional literacy, may mitigate the adverse effects of cirrhosis and promote positive changes in dietary behaviors and QoL. Furthermore, the combined use of visual and printed educational materials, as opposed to relying on a single method, may enhance the effectiveness of nutrition education interventions. Moreover, integrating mobile health technologies (e.g., evidence-based mobile applications), establishing peer support networks to foster social support and awareness, developing targeted behavior change models, organizing group-based training sessions, and providing professional guidance are strategies that may further improve the efficacy of educational interventions aimed at enhancing nutritional and health literacy among patients.

Due to the complex pathophysiology of liver cirrhosis, which impacts multiple metabolic processes and the frequent presence of comorbidities, polypharmacy, and sociodemographic or psychological factors, it is essential to consider these elements comprehensively in both nutritional evaluations and the development of individualized educational interventions. Furthermore, the high prevalence of concurrent chronic conditions and the potential for nutrient–drug interactions should be carefully considered when planning dietary therapies for this population. Future high-quality research with larger study groups and longer follow-ups is warranted to further explore the effects of nutritional education on the nutritional knowledge, nutritional status, and QoL in patients with liver cirrhosis.

## Figures and Tables

**Table 1:** Socio-demographic characteristics and anthropometric measurements of participants.

	Female (n = 9)		Male (n = 21)		Total (n = 30)		*p* *
	$(\bar{X}$ ± SD)		$(\bar{X}$ ± SD)		$(\bar{X}$ ± SD)		
Age (years)	43.1 ± 9.45		46.0 ± 11.55		45.1 ± 10.88		0.515 ^#^
Time since diagnosis (years)	3.4 ± 2.03		2.9 ± 1.91		3.1 ± 1.93		0.164
BMI (baseline) (kg/m^2^)	26.0 ± 4.63		27.7 ± 5.43		26.6 ± 5.03		0.288
BMI (at T1) (kg/m^2^)	26.2 ± 4.61		28.0 ± 5.82		27.1 ± 5.22		
MUAC (baseline) (cm)	26.9 ± 4.26		29.1 ± 3.90		28.0 ± 4.08		0.841
MUAC (at T1) (cm)	26.9 ± 4.31		29.0 ± 5.00		27.95 ± 4.66		
TSF (baseline) (mm)	24.1 ± 10.01		21.2 ± 9.22		22.7 ± 9.62		0.543
TSF (at T1) (mm)	23.9 ± 9.73		20.7 ± 10.41		20.3 ± 10.07		
	n	%	n	%	n	%	
Education level							
Primary–secondary	6	66.7	7	33.4	13	43.3	0.101
High school	3	33.3	10	47.6	13	43.3	
University	-	-	4	19.0	4	13.4	
Cause of liver cirrhosis							
Chronic hepatitis B	2	22.2	9	42.8	11	36.7	0.518
Cholestatic diseases	5	55.6	5	23.8	10	33.3	
Alcohol-related cirrhosis	-	-	3	14.3	3	10.0	
Non- alcoholic steatohepatitis	-	-	3	14.3	3	10.0	
Cryptogenic	2	22.2	1	4.8	3	10.0	
Comorbidities							
Cardiovascular diseases	2	25.0	2	50.0	4	33.2	1.000
Respiratory diseases	1	12.5	1	25.0	2	16.7	1.000
Hematologic diseases	1	12.5	1	25.0	2	16.7	1.000
Thyroid diseases	2	25.0	-	-	2	16.7	1.000
Bone and joint diseases	2	25.0	-	-	2	16.7	1.000
Regular physical activity ^b^							
Yes	3	33.3	9	42.9	12	40.0	1.000
No	6	66.7	12	57.1	18	60.0	

*: chi-square test; #: independent samples *t*-test; X: mean; SD: standard deviation; n: number; BMI: body mass index; T1: at the end of the treatment; MUAC: mid-upper-arm circumference; TSF: triceps skinfold thickness; b: 150 min/week of moderate-intensity physical activity.

**Table 2:** Biochemical findings of the participants before and after the nutritional education.

	Female (n = 9)				Male (n = 21)				p3 ^#^
	Before Education ($\bar{X}$ ± SD)	After Education ($\bar{X}$ ± SD)	Change ∆ (%)	p1 ^&^	Before Education ($\bar{X}$ ± SD)	After Education ($\bar{X}$ ± SD)	Change ∆ (%)	p2 ^&^	
Albumin (g/L)	40.4 ± 5.10	40.1 ± 5.51	−0.3 (0.7)	0.235	34.8 ± 9.34	34.1 ± 8.85	−0.7 (2.0)	0.205	0.965
Hemoglobin (g/dL)	12.7 ± 1.30	12.6 ± 2.19	−0.1 (0.8)	0.854	12.1 ± 2.66	11.9 ± 2.83	−0.2 (1.7)	0.517	0.625
Glucose (mg/dL)	89.1 ± 14.32	87.2 ± 9.74	−1.9 (2.3)	0.743	110.3 ± 58.70	96.4 ± 20.46	−13.9 (12.6)	0.263	0.929
Total protein (g/L)	70.0 ± 7.11	69.1 ± 5.67	−0.9 (1.3)	0.655	64.6 ± 9.54	67.4 ± 8.42	+2.8 (4.3)	0.293	1.000
GGT (U/L)	126.0 ± 211.23	106.8 ± 157.82	−19.2 (15.2)	0.034 *	76.0 ± 78.88	55.2 ± 37.67	−20.8 (27.4)	0.018 *	0.790
AST (U/L)	93.7 ± 129.93	59.0 ± 55.51	−34.7 (37.3)	0.021 *	65.8 ± 55.32	54.9 ± 27.59	−10.9 (16.6)	0.338	0.114
ALT (U/L)	69.4 ± 80.55	49.3 ± 35.79	−20.1 (28.9)	0.048 *	61.1 ± 87.91	42.6 ± 19.23	−18.5 (30.3)	0.012 *	0.244
Total-C (mg/dL)	175.9 ± 44.85	177.2 ± 57.66	+1.3 (0.7)	0.900	139.8 ± 58.43	144.0 ± 50.50	+4.2 (3.0)	0.631	0.894
LDL-C (mg/dL)	106.7 ± 31.35	120.9 ± 59.91	+14.2 (13.3)	0.228	81.0 ± 38.21	76.0 ± 35.24	−5.0 (6.2)	0.457	0.070
HDL-C (mg/dL)	44.3 ± 17.47	37.1 ± 15.85	−7.2 (16.3)	0.248	37.2 ± 24.76	47.2 ± 26.65	+10 (26.9)	0.046 *	0.086
Triglycerides (mg/dL)	121.7 ± 153.08	99.4 ± 35.60	−22.3 (18.3)	0.025 *	107.9 ± 43.90	102.3 ± 77.93	−5.6 (5.2)	0.710	0.025 *
VLDL-C (mg/dL)	24.3 ± 30.61	19.3 ± 7.71	−5.0 (20.6)	0.579	21.6 ± 8.87	21.6 ± 16.67	0.0	1.000	0.094
Non-HDL (mg/dL)	132.1 ± 47.28	140.1 ± 64.89	+8.0 (6.1)	0.605	98.6 ± 48.04	154.6 ± 276.37	+56.0 (56.8)	0.356	0.504
Vit D (nmol/L)	39.1 ± 22.73	39.2 ± 34.55	+0.1 (0.3)	0.994	39.1 ± 16.73	44.4 ± 21.0	+5.3 (13.6)	0.214	0.137
Vit B12 (pg/mL)	443.8 ± 135.24	383.9 ± 189.70	−59.9 (13.5)	0.258	651.3 ± 389.14	726.6 ± 443.04	+75.3 (11.6)	0.195	0.137
Sodium (mEq/L)	140.6 ± 2.92	201.7 ± 184.65	+61.1 (43.5)	0.720	138.7 ± 3.23	137.4 ± 3.60	−1.3 (0.9)	0.111	0.349
Potassium (mEq/L)	4.1 ± 0.30	4.0 ± 0.23	−0.1 (2.4)	0.103	4.2 ± 0.36	4.3 ± 0.38	+0.1 (2.4)	0.419	0.125
Phosphorus (mg/dL)	3.3 ± 0.42	3.4 ± 0.51	+0.1 (3.0)	0.722	3.2 ± 0.64	3.3 ± 0.53	+0.1 (3.1)	0.432	0.756
Calcium (mg/dL)	9.2 ± 0.68	9.1 ± 0.51	−0.1 (1.1)	0.628	8.6 ± 0.60	8.7 ± 0.68	+0.1 (1.2)	0.423	0.326
Magnesium (mg/dL)	1.8 ± 0.16	1.8 ± 0.17	0.0	1.000	1.7 ± 0.24	1.8 ± 0.24	+0.1 (5.9)	0.100	0.397

&: dependent sample *t*-test; #: Mann–Whitney U test; * *p* < 0.05; p1: p between females; p2: p between males; p3: p between genders; GGT: gamma glutamyl transferase; AST: aspartate aminotransferase; ALT: alanin aminotransferase; Total-C: total cholesterol; LDL:C: low-density lipoprotein cholesterol; HDL-C: high-density lipoprotein cholesterol; VLDL-C: very low-density lipoprotein cholesterol; mg: milligram; g: gram; L: liter; dL: desiliter; U: unit; mEq: milliequivalent; pg: picogram; X: mean; SD: standard deviation. Biochemical assessments were conducted at two time points: one week prior to the initial nutrition education session and within one week after the end of the three-month nutritional education.

**Table 3:** Total energy and macronutrient intakes of participants before and after the nutritional education.

	Female (n = 9)			Male (n = 21)		
	Before Education ($\bar{X}$ ± SS)	After Education ($\bar{X}$ ± SS)	p1 *	Before Education ($\bar{X}$ ± SS)	After Education ($\bar{X}$ ± SS)	p2 *
TEE (kcal)	1293.3 ± 511.20	1064.6 ± 631.31	0.069	1634.2 ± 562.02	1638.5 ± 512.98	0.981
TEE (kcal/kg)	20.9 ± 11.19	16.9 ± 11.40	0.005 *	21.3 ± 9.95	21.2 ± 8.13	0.089
CHO (g)	169.2 ± 77.69	110.5 ± 83.16	0.017 *	193.2 ± 75.10	181.2 ± 51.61	0.569
CHO (%TEE)	52.9 ± 10.27	39.9 ± 12.19	0.023 *	49.3 ± 10.77	46.3 ± 7.43	0.266
Protein (g)	37.3 ± 15.50	34.8 ± 17.93	0.532	59.7 ± 30.60	62.3 ± 28.03	0.760
Protein (g/kg)	0.6 ± 0.38	0.5 ± 0.31	0.023 *	0.7 ± 0.56	0.8 ± 0.40	0.040 *
Protein (%TEE)	11.9 ± 2.37	14.0 ± 2.60	0.072	14.9 ± 5.92	15.1 ± 3.18	0.813
Plant protein (g)	26.0 ± 11.92	20.9 ± 16.58	0.235	28.0 ± 10.60	29.9 ± 12.16	0.615
Fat (g)	50.6 ± 24.86	52.6 ± 28.55	0.736	67.1 ± 31.98	71.7 ± 28.25	0.618
Fat (%TEE)	35.3 ± 10.24	46.1 ± 10.59	0.043 *	35.8 ± 8.89	38.5 ± 7.05	0.246
Cholesterol (mg)	152.6 ± 104.47	190.9 ± 177.23	0.418	319.1 ± 257.54	284.9 ± 233.56	0.508
SFA (%TEE)	14.4 ± 6.39	19.4 ± 3.53	0.102	15.1 ± 4.45	16.5 ± 3.95	0.300
MUFA (%TEE)	11.8 ± 3.93	16.2 ± 5.33	0.115	12.4 ± 3.96	14.1 ± 3.63	0.147
PUFA (%TEE)	6.7 ± 2.84	7.7 ± 5.72	0.643	6.3 ± 3.06	5.6 ± 2.60	0.375
Fiber (g)	13.9 ± 6.29	11.1 ± 5.88	0.289	17.6 ± 8.00	18.6 ± 8.80	0.701
Water (mL/d) ($\bar{X}$ ± SS)	1844.4 ± 1142.49	2083.3 ± 1346.29	0.835	1690.5 ± 1016.32	1704.7 ± 679.50	0.670

* dependent sample *t*-test, *p* < 0.05; p1: p between females; p2: p between males; TEE: total energy expenditure; CHO: carbohydrate; SFA: saturated fatty acids; MUFA: monounsaturated fatty acids; PUFA: polyunsaturated fatty acids; X: mean; SD: standard deviation.

**Table 4:** Micronutrient intakes of participants before and after the nutritional education.

	Female (n = 9)			Male (n = 21)		
	Before Education$\;\bar{X}$ ± SS (%RDA ^#^)	After Education$\;\bar{X}$ ± SS (%RDA)	p1 *	Before Education$\;\bar{X}$ ± SS (%RDA)	After Education$\;\bar{X}$ ± SS (%RDA)	p2 *
Vitamins						
Vitamin A (mcg)	556.5 ± 431.36 (79.5)	692.4 ± 536.84 (98.9)	0.440	809.1 ± 600.55 (89.9)	745.4 ± 246.99 (82.8)	0.649
Vitamin E (mg)	7.8 ± 4.77 (52.3)	8.9 ± 8.62 (59.5)	0.737	13.5 ± 10.76 (90.0)	11.7 ± 8.09 (77.7)	0.553
Rhiamine (mg)	0.5 ± 0.19 (42.7)	0.5 ± 0.23 (40.9)	0.829	0.7 ± 0.33 (60.8)	0.9 ± 0.32 (73.3)	0.153
Riboflavin (mg)	0.7 ± 0.22 (64.5)	0.8 ± 0.49 (72.7)	0.542	1.4 ± 0.84 (103.8)	1.4 ± 0.56 (108.5)	0.773
Niacin (mg)	6.9 ± 2.98 (49.1)	5.6 ± 3.15 (40.1)	0.381	12.2 ± 10.82 (76.1)	12.0 ± 8.48 (75.0)	0.926
Folic acid (mcg)	162.5 ± 76.53 (40.6)	185.1 ± 135.08 (46.3)	0.540	237.1 ± 89.51 (59.3)	267.5 ± 113.70 (66.9)	0.324
Vitamin B6 (mg)	0.6 ± 0.23 (49.2)	0.6 ± 0.34 (50.0)	0.953	1.0 ± 0.42 (73.1)	1.2 ± 0.63 (88.5)	0.255
Vitamin B12 (mcg)	1.6 ± 0.66 (68.3)	2.4 ± 1.57 (100.4)	0.085	4.3 ± 4.90 (176.3)	4.3 ± 2.60 (180.4)	0.929
Vitamin C (mg)	39.4 ± 32.73 (52.6)	63.5 ± 61.51 (84.6)	0.321	79.8 ± 65.99 (88.6)	84.9 ± 66.02 (94.3)	0.815
Minerals						
Potassium (mg)	1101.5 ± 429.12 (42.4)	1307.5 ± 831.10 (50.3)	0.458	2092.5 ± 963.47 (61.5)	2232.3 ± 841.23 (65.7)	0.621
Calcium (mg)	408.3 ± 188.81 (40.8)	463.7 ± 302.75 (46.4)	0.560	585.4 ± 278.9 (58.5)	735.8 ± 397.60 (73.6)	0.195
Phosphorus (mg)	616.9 ± 166.4 (88.1)	579.5 ± 315.35 (82.8)	0.697	931.0 ± 420.40 (133.0)	1045.2 ± 415.1 (149.3)	0.342
Iron (mg)	5.6 ± 2.03 (69.1)	5.2 ± 2.61 (65.5)	0.730	8.0 ± 4.9 (109.9)	8.4 ± 3.74 (104.6)	0.732
Zinc (mg)	5.6 ± 1.65 (70.5)	5.6 ± 3.11 (70.3)	0.978	8.8 ± 6.26 (110.1)	9.4 ± 3.83 (117.3)	0.742
Magnesium (mg)	154.0 ± 39.98 (48.1)	136.7 ± 73.41 (42.7)	0.485	210.6 ± 77.55 (50.1)	238.3 ± 99.31 (56.7)	0.364
Copper (mcg)	931.3 ± 396.99 (103.5)	704.4 ± 328.18 (78.3)	0.058	1271.9 ± 689.48 (141.3)	1140.0 ± 429.52 (126.7)	0.414

* dependent sample *t*-test, *p* < 0.05; p1: p between females; p2: p between males; RDA: recommended dietary allowance; #: percentage of RDA met; mcg: microgram; mg: milligram; X: mean; SD: standard deviation.

**Table 5:** Classification using Subjective Global Assessment of individuals.

	Female (n = 9)				Male (n = 21)				Total (n = 30)			
	Before Education		After Education		Before Education		After Education		Before Education		After Education	
	n	%	n	%	n	%	n	%	n	%	n	%
A score	3	33.3	5	55.6	8	38.1	13	61.9	11	36.7	18	60.0
B score	5	55.6	4	44.4	11	52.4	7	33.3	16	53.3	11	36.7
C score	1	11.1	-	-	2	9.5	1	4.8	3	10.0	1	3.3
*p* *	1.000				0.142				0.065			

* McNemar test, *p* < 0.05; SGA: Subjective Global Assessment; A score: no malnutrition; B score: mild–moderate malnutrition; C score: Severe malnutrition.

**Table 6:** Quality of life and nutritional knowledge level scale scores of individuals before and after nutrition education.

	Female (n = 9)				Male (n = 21)				Total (n = 30)			
SF-12 scores												
PCS-12	41.2 ± 10.55		48.0 ± 12.33		43.8 ± 11.21		43.7 ± 8.99		43.0 ± 10.90		45.0 ± 10.09	
Change, ∆ (%)	+6.8 (16.5)				−0.1 (0.2)				+2.0 (4.7)			
*p* *	0.211				0.947				0.369			
MCS-12	41.9 ± 10.94		43.1 ± 12.24		50.9 ± 11.64		46.3 ± 9.49		48.2 ± 12.00		45.3 ± 10.28	
Change, ∆ (%)	+1.2 (2.9)				−4.6 (9.0)				−2.9 (6.0)			
*p* *	0.810				0.035 *				0.156			
NKL score	6.3 ± 1.94		10.0 ± 3.64		7.9 ± 2.97		8.9 ± 3.40		7.4 ± 2.76		9.2 ± 3.45	
Change, ∆ (%)	+3.7 (58.7)				+1.0 (12.7)				+1.8 (24.3)			
*p* *	0.008 *				0.116				0.004 *			
NKL groups	n	%	n	%	n	%	n	%	n	%	n	%
Low NKL	3	33.3	1	11.1	4	19.0	3	14.3	7	23.3	4	13.3
Mid NKL	6	66.7	8	88.9	16	76.2	16	76.2	22	73.3	24	80.0
High NKL	-	-	-	-	1	4.8	2	9.5	1	3.3	2	6.7
*p* ^#^	0.625				0.565				0.403			

* sependent sample *t*-test; #: McNemar test, *p* < 0.05; NKL: nutrition knowledge level; SF-12: quality of life scale; PCS-12: physical component summary; MCS-12: mental component summary; low NKL: ≤5 score; mid NKL: 6–15 score; high NKL: ≥15 score.

## Data Availability

The data presented in this study are available on request from the corresponding author due to personal data protection.

## Abbreviations

The following abbreviations are used in this manuscript:

BMI	Body mass index
DRI	Dietary reference intake
SGA	Subjective Global Assessment
QoL	Quality of life
SF-12	The 12-item short-form health survey
PCS-12	Physical component summary
MCS-12	Mental component summary
NKL	Nutritional Knowledge Level
GGT	Gamma-glutamyl transferase
AST	Aspartate aminotransferase
ALT	Alanine aminotransferase
RDI	Recommended dietary intake
TUBER	Turkey Dietary Guidelines
ESPEN	The European Society for Clinical Nutrition and Metabolism
EASL	The European Association for the Study of the Liver
